# The intrinsic reason why the term “central sensitization” should be refined, by introducing the terms “spinal sensitization”, “pain-related brain network sensitization”, and “psychosocial-related sensitization”

**DOI:** 10.3389/fpain.2026.1825371

**Published:** 2026-08-21

**Authors:** Jean-Pascal Lefaucheur

**Affiliations:** 1 UR4391 (ENT Team), Faculty of Health, Paris Est Créteil University, Créteil, France; 2 Department of Clinical Neurophysiology, Henri Mondor University Hospital, AP-HP, Créteil, France

**Keywords:** brain networks, chronic pain, diagnosis, model, psychosocial factors, sensitization, spinal cord

## 1 Introduction

Acute pain occurs in response to a noxious stimulus and serves as a warning signal linked to a threat to the body, prompting a protective reaction. In cases of chronic pain involving nociceptive mechanisms, such as those associated with chronic inflammatory joint diseases, pain may continue to play a protective role by limiting use of the affected body part and promoting healing. In other cases, such as those involving neuropathic or nociplastic mechanisms, chronic pain most likely does not result from persistent noxious stimuli and can be considered maladaptive. Thus, in many situations, the transition to chronic pain can be explained by molecular and cellular sensitization processes. These sensitization processes can occur at the level of peripheral receptors, primary afferent sensory fibers, spinal synaptic integration, or somatosensory pathways within the central nervous system (1). In particular, in nociplastic pain, there is no direct link between a traumatic event (or its severity) and the onset or persistence of pain and central sensitization processes are considered to play a key role (2). However, there is considerable ambiguity regarding the term “central sensitization”.

According to the International Association of Pain (IASP), sensitization is defined as an “increased responsiveness of nociceptive neurons to normal or subthreshold afferent inputs” (3, 4), occurring in the peripheral or central nervous system. Thus, the IASP considers “central sensitization” to be rather a “neurophysiological term”, explaining pain amplification due to enhanced neuronal responsiveness, clinically corresponding to “evoked pain” phenomena, such as hyperalgesia or possibly allodynia. This conception, which corresponds to the first experimental descriptions of post-injury central sensitization to pain (5), is however restrictive. Indeed, in routine clinical practice, the term “central sensitization” encompasses signs and symptoms that are by no means limited to hyperalgesia and allodynia. For example, in one of the most widely used questionnaires to clinically assess “central sensitization”, the “Central Sensitization Inventory” (CSI) (6), there are diagnostic items with (i) a cognitive valence, such as “I have difficulty concentrating” or “I have difficulty remembering things”, (ii) an affective valence, such as “I have anxiety attacks”, or “I feel sad or depressed”, or (iii) a psychosocial valence, such as “I suffered trauma as a child” or “I need help in performing my daily activities”. Thus, the term “central sensitization” is used to define very different clinical aspects and pathophysiological mechanisms, reflecting the fact that chronic pain syndrome is a multidimensional experience.

A clinical picture labeled “central sensitization” can correspond to three main pathophysiological mechanisms: (i) spinal (dorsal horn) sensitization; (ii) pain-related brain network sensitization, possibly including pain modulatory system dysfunction; (iii) psychosocial-related sensitization. The term “central sensitization” is therefore too vague to be informative, and this is the intrinsic reason why it should be refined based on the different mechanisms involved, which will be briefly discussed in this article. Such an approach would also facilitate clinical phenotyping according to the corresponding pathophysiological mechanisms.

First, sensitization may occur at the peripheral level, secondary to persistent activation of nociceptors by inflammatory mediators in the context of nociceptive pain. Sensitization may then be related to changes in membrane expression or trafficking of nociceptors or in the thresholds and activation kinetics of associated ion channels (7, 8). This can lead to increased axonal excitability or altered intra-axonal signal transduction. Other “peripheral” sensitization processes include the recruitment of “silent” nociceptors (9), “gain-of-function” changes in sensory afferents (10), or their “reprogramming” resulting from a phenotypic switch (11) or local cross-talk mechanisms (12), not to mention the role of non-neuronal cells such as keratinocytes (13). The axon initial segment (14) or the cell body (15) of first-order sensory neurons in the dorsal root ganglia can also be considered the primary sites of peripheral sensitization.

Thus, peripheral nerve sensitization may be either a secondary process to abnormal or prolonged activation of nociceptors in nociceptive pain, or a primary process due to axonal or neuronal disorder in peripheral neuropathic pain. Clinically, beyond increased spontaneous activity, this manifests as hyperalgesia, which is an intensification of pain caused by a normally painful stimulus.

## 2 Spinal (dorsal horn) sensitization

Hyperalgesia may also reveal a central sensitization process occurring in the dorsal horn of the spinal cord (7, 8). For the facial region, the corresponding central structure is the trigeminal sensory nuclear complex located in the brainstem (16). Therefore, in this case, one must obviously refer to “brainstem” rather than “spinal” sensitization, but molecular and cellular mechanisms are similar to those involved in spinal sensitization.

A distinction must also be made between “primary” and “secondary” hyperalgesia (8). Primary hyperalgesia occurs in the anatomical territory of damaged non-neural or neural tissues (homotopic phenomenon), and is therefore the spinal counterpart of peripheral sensitization. Secondary hyperalgesia is characterized by increased pain sensitivity in undamaged tissues surrounding the lesion (heterotopic phenomenon). Multiple mechanisms can contribute to the phenomenon of secondary hyperalgesia, ranging from peripheral mechanisms, such as distal axon reflexes (17) and the expansion of receptive fields, to mostly central mechanisms with convergent projections and synaptic plasticity processes within the dorsal horns, characterized by reduced inhibition and the awakening of “silent” neuronal networks, leading to increased responsiveness of spinal nociceptive neurons (18, 19). Secondary hyperalgesia can be assessed by various neurophysiological protocols, mainly based on the recording of evoked potentials or nociceptive reflexes (20, 21). Quantitative sensory testing can also reveal abnormal synaptic changes in the spinal cord in the context of hyperalgesia, based on temporal summation or wind-up protocols. However, beyond explaining some specific clinical conditions of “evoked pain”, these protocols cannot provide information on spontaneous/ongoing neuropathic pain.

At the level of the dorsal horns, other changes in synaptic plasticity can cause allodynia, which is a pain evoked by a normally non-painful stimulus. Abnormal reactivity of non-nociceptive Aβ or Aδ/C fibers produces mechanical or thermal allodynia, respectively. The pathophysiological mechanisms underlying allodynia may also involve a reduction in the inhibitory control exerted over “silent” neuronal spinal connections, leading to the transmission of low-threshold information to nociceptive pathways (22). This phenomenon thus relies on changes in interactions between non-nociceptive afferents and nociceptive projection neurons, involving various types of dorsal horn neurons, such as those expressing calretinin or protein kinase C gamma, in the case of mechanical allodynia of nociceptive or neuropathic origin, respectively (23).

## 3 Pain-related brain network sensitization

### 3.1 Pain matrices

A key concept in brain neurophysiology is to view the network, rather than the brain region, as the functional unit (24, 25). Thus, even if a pathology is assumed to primarily alter a specific brain structure, the entire network to which that structure belongs is affected. It is precisely the altered functioning of this network that is associated with the clinical symptomatology. In the context of pain-related central sensitization, the alteration of brain networks is based on the concept of the existence of three “pain matrices” (26).

The first-order pain matrix (“nociceptive”, “sensations of pain”) includes the superior-posterior insula, the parietal operculum, the mid-cingulate cortex, and the postcentral gyrus. This matrix is involved in sensory encoding. Its activation produces a variety of neuropathic-like sensations (27). The second-order pain matrix (“cognitive”, “attention to pain”) includes the anterior cingulate cortex, the dorsal anterior insula, the dorsolateral prefrontal cortex, and the posterior parietal cortex. This matrix is involved in attentional modulation. Its activation compromises cognitive reserve (28) and impairs adaptation to activities of daily living. The third-order pain matrix (“affective”, “emotions related to pain”) includes the perigenual cingulate cortex, the orbitofrontal cortex, and the anterolateral prefrontal cortex. This matrix is involved in the regulation of emotions. Its activation is associated with various negative emotions and poor self-image. Altered intra-/inter-matrix functional connectivity may be involved in neuropathic pain, even of peripheral origin (29).

This division into three distinct networks may appear somewhat artificial, particularly regarding the functional specialization of brain regions viewed as either “cognitive” or “affective”. However, within the anterior insula, one can distinguish a ventral part associated with socio-emotional components and a dorsal part associated with cognitive components (30). The dorsal anterior insula contributes to response inhibition, task switching, and information updating, as well as to the regulation of executive control and working memory. It also plays a role in salience processing, attention, and gatekeeping for conscious awareness. Another example concerns the prefrontal cortical region. The dorsolateral prefrontal cortex (DLPFC) is primarily involved in executive functions such as planning, decision-making, working memory, personality expression, and the regulation of social behavior (31). Major depressive disorder is associated with DLPFC dysfunction, partly because the disorder often involves deficits in cognitive control and because connections exist between emotional and cognitive networks. However, the DLPFC is not directly involved in affective components such as sadness, anhedonia, or feelings of hopelessness. These symptoms instead involve other prefrontal regions, notably the orbitofrontal cortex (32).

However, this highly spatialized view is challenged in complex situations that combine cognition and emotion and rely on dynamic interactions between various brain region networks (33, 34). At the heart of these interactions between cognition and emotion lie highly connected brain regions, known as “hubs” or key nodes, that play a crucial role in multimodal information integration. One example of such a structure is the amygdala, which may act as a key structure in the processes of “pain-related brain network sensitization”. Indeed, several recent publications position the amygdala as a critical “hub” at the interface between cognitive and affective networks, rather than as belonging specifically to one or the other, in the context of chronic pain (35, 36).

### 3.2 Other brain network models

Two other “triple” brain network models have been proposed as being involved in central pain sensitization. First is the “canonical” triple-network model, comprising the default mode network (DMN), the salience network (SN), and the central executive network (CEN, or frontoparietal network, FPN). This model has been extensively studied to explain large-scale brain dynamics, particularly in the context of psychiatric and cognitive disorders as well as the relationship between depression and cognitive impairment (37–39). The DMN-SN-CEN/FPN model has also been proposed to play a key role in chronic pain syndromes (40, 41). However, a variant of this model has been suggested to be more specifically involved in the context of chronic pain, including the sensorimotor network (SMN) instead of the CEN/FPN (2).

The DMN comprises the medial prefrontal cortex, the posterior cingulate cortex, the precuneus, and the middle temporal gyrus. This network is involved in self-referential thinking and is generally active at rest. The SN connects the dorsal anterior cingulate cortex and the anterior insula. This network is involved in attentional modulation and the integration of various environmental inputs. The CEN/FPN connects the DLPFC and the posterior parietal cortex. This network is involved in sustained attention, complex problem-solving, and working memory. The SMN connects the primary sensory and motor cortices. This network participates in the processing of sensory inputs and the motor response. Interestingly, the primary motor cortex is classically considered a prime target for cortical stimulation techniques aimed at treating chronic pain syndromes (42). However, as a “motor” structure, the primary motor cortex likely does not play a critical role in processing pain-related information, specifically. In fact, the primary motor cortex is located in the precentral gyrus, a crossroad cortical region now known to contain non-motor, inter-effector regions involved in large-scale cognitive networks (43). The modulation of these networks, rather than the activation of motor structures themselves, likely explains the efficacy of precentral cortical stimulation in treating chronic pain syndromes (44).

Dysfunction in the triple-network system (DMN-SN-CEN/FPN or SMN) may explain disruptions in the dynamic coordination of information processing regarding self-referential processing, salient experiences, attention to external stimuli, emotional arousal, autonomic regulation, and the integration of sensory bodily signals. Although such dysfunction may play a role in the development of chronic pain, these models focus primarily on cognition and interoception. Consequently, they appear more closely linked to causal phenomena than to the clinical symptomatic expression of the central pain sensitization process. In this regard, the triple-network model based on “pain matrices” shows a clearer association with the pathophysiological mechanisms underlying the nociceptive, cognitive, and affective components of chronic pain syndrome and sensitization.

### 3.3 Pain modulatory system dysfunction

According to the IASP definition (3, 4), central sensitization can also result from “a dysfunction of endogenous pain control systems”. The main control system is the “descending pain modulatory system” (DPMS), which exerts either an inhibitory or a facilitatory effect on the transmission of pain signals (45, 46). This is a purely “top-down” control mechanism, triggered in response to the activation of higher cortical centers, whether cognitive or affective, by afferent nociceptive inputs. Thus, most of the brain structures involved in the pain matrices presented previously can interact critically with the DPMS. The DPMS pathways, originating from cortical and subcortical-thalamic structures, relay through various brainstem structures: the periaqueductal gray (PAG), a mesencephalic structure; the locus coeruleus (LC), a posterolateral pontine structure; the nucleus raphe magnus (NRM) and the rostral ventromedial medulla (RVM), pontomedullary structures; and the dorsal reticular subnucleus (SRD, also known as the dorsal reticular nucleus), a posterior medullary structure. Activation of this system involves various neurotransmitter systems, notably enkephalinergic, noradrenergic, and serotonergic. The final action of DPMS is to modulate the transmission of the nociceptive signal within the dorsal horns of the spinal cord. The DPMS therefore lies at the interface between the activation by brain structures belonging to pain matrices and the spinal sensitization that occurs in the dorsal horns.

Another pain modulation system relies on the activation of various DPMS structures located in the brainstem by heterotopic nociceptive inputs (originating from an area other than the primary pain site). This mechanism, which operates via a spinal-brain-spinal loop, is known as “diffuse noxious inhibitory control” (DNIC), a phenomenon first demonstrated experimentally in rats (47, 48). This system, which underlies the phenomenon of “pain inhibition by pain”, should not be confused with the “top-down” control exerted by cortical centers via the DPMS, even though they share many common projection structures within the brainstem. DNIC can be clinically assessed using “conditioned pain modulation” (CPM) protocols (49, 50). Although CPM protocols likely reflect modulation processes that are more complex than a simple DNIC phenomenon, they probably fail to fully capture cortically driven DPMS control mechanisms. Furthermore, since these protocols rely on experimentally induced pain methods, they can only imperfectly reflect the role that potential dysfunction of these control mechanisms might play in the context of spontaneous chronic pain.

Impairment of inhibitory DPMS control could, under certain conditions, contribute to spontaneous pain states. However, it primarily leads to a substantial amplification, facilitation, or unmasking of nociceptive signal transmission, potentially involved in the chronification of pain. Beyond abnormal responses to CPM protocols, DPMS dysfunction is complex to define and delineate in terms of clinical symptoms compared to the implication of the triple network model of pain matrices, which is characterized by sensory, cognitive, or affective symptomatology.

## 4 Psychosocial-related sensitization

Chronic pain syndrome is not limited to changes in the activity of sensory neurons and pathways. It is a multidimensional experience (sensory, cognitive, and affective) influenced, to varying degrees, by psychological vulnerability and social stressors linked to the patient's personal and family history. These factors can fuel the vicious cycle (or spiral) linking pain, cognitive impairment, stress, anxiety, depression, and impaired daily functioning, including disrupted physical activity and chronic fatigue (51). This vicious cycle reinforces alterations in the connectivity and reactivity of brain networks, affecting both the integration of incoming afferent information and the generation of outgoing efferent messages and resulting behaviors.

The psychosocial vulnerability-stress (or diathesis-stress) model was initially proposed to explain schizophrenia (52). This theory posits that the impact of an initial triggering life event (personal, social, or environmental), experienced as a stressor, is more likely to contribute to disability in individuals with a pre-existing biological or psychological predisposition (vulnerability) to be preoccupied with their bodily or interoceptive cues (hyperawareness) or to react with fear to the implications of perceived symptoms (catastrophizing). These individuals are at increased risk of disability amplification and persistence. Actually, they are more prone to central sensitization of a symptom in response to environmental influences (stressors) due to their pre-existing vulnerability. This model has also been applied to explain chronic pain syndromes (53, 54). Factors that contribute to vulnerability to pain include genetic predispositions, neurodevelopmental abnormalities, a history of physical trauma that may have sensitized the nervous system, poor general health, low self-esteem, catastrophizing, maladaptive beliefs, certain personality traits, early psychological trauma, insufficient cognitive reserve, social isolation, or negative life events. Conversely, there are protective factors that act as a buffer and mitigate vulnerability, such as resilience, coping strategies, positive operant conditioning, or strong social support.

It is clear that psychosocial vulnerability and stress factors can ultimately strengthen the central sensitization mechanisms involving pain-related brain networks. However, the networks associated with these psychosocial factors, even if they share some brain structures with pain-related networks, have certain structural and organizational differences and can operate independently. Attempts to identify the networks involved in psychosocial vulnerability to painful conditions have been reported (54), particularly highlighting the reward-motivation learning network and the DPMS. Networks linking psychological stressors and physical health, and potentially involved in stress-induced pain sensitization, are also known (55). These include the sympatho-adreno-medullary (SAM) and hypothalamic-pituitary-adrenal (HPA) axes, which involve neuroendocrine and immune regulation, as well as brain networks connecting the medial prefrontal cortex and the anterior and mid-cingulate cortices, and various subcortical structures, such as the PAG and the thalamus, for example. A particular domain of stress scenarios relates to social factors. Brain networks supporting social cognition processes have also been identified in humans (56), as well as those involved in the relationship between social stress and pain vulnerability (57). Some authors have previously proposed incorporating a social component into the definition of chronic pain (58), but this proposal was not adopted (59). It is essential to consider this social dimension when evaluating chronic pain syndrome and sensitization to pain, as it can be a decisive factor in therapeutic management. Indeed, social support can have beneficial effects by alleviating the suffering of patients with chronic pain, although certain forms of emotional or material aid, such as excessive solicitude or financial assistance, can also lead to adverse consequences (60).

Thus, sensitization mechanisms related to psychosocial vulnerability and stress factors involve brain networks that do not have a specific role in the processing or generation of nociceptive information, unlike those involved in “pain-related brain network sensitization”, associated with the sensory, cognitive, and affective aspects of chronic pain syndrome. The identification of psychosocial difficulties or risk factors in an individual may or may not play a role in the development of such a syndrome. Only the analysis of clinical elements will allow to suggest that the combination of stressors and predisposing psychosocial vulnerability is significant enough to contribute to pain sensitization. Similarly, if a lesion or disease of the nervous system is discovered in a patient suffering from pain, it is not possible to automatically attribute a causal link to the pain, nor to conclude that a neuropathic pain syndrome exists. Such a conclusion will be based solely on the interpretation of all clinical elements, primarily the profile of painful symptoms.

## 5 Conclusion

The term “central sensitization” is common and widely used, but it needs clarification because it is too vague and confusing, reflecting many different pathophysiological mechanisms. For example, central sensitization can be linked to an abnormal interaction between non-nociceptive Aβ and Aδ/C fibers and nociceptive projection neurons in the dorsal horns of the spinal cord. These neurophysiological mechanisms of spinal sensitization reflect an increased responsiveness of neurons that underlies the original definition of central sensitization according to the IASP. This can explain evoked pain symptoms (hyperalgesia/allodynia), but not the diffuse pain observed in nociplastic pain, as assessed by the CSI questionnaire, which is associated with another type of central sensitization. In this case, central sensitization is linked to abnormal connectivity or maladaptive plasticity within various brain networks, explaining diffuse and polymorphic sensory symptoms and bodily hypersensitivity associated with a significant burden of cognitive and affective disturbances. Alterations in intra/interconnective connectivity or dysfunction of various pain-related brain networks, underlying this type of central sensitization, can be associated with abnormal dysfunction of the endogenous pain control systems (DPMS, DNIC). These alterations could constitute the mechanism underlying primary nociplastic pain, in the absence of peripheral nociceptor activation or alteration of somatosensory pathways. On the other hand, these alterations could represent a secondary mechanism involved in the chronification of pain, in its multidimensional aspects and its interference with activities of daily living, in cases of initially nociceptive or neuropathic pain syndrome. The chronicity of pain is then accompanied by changes in clinical presentation, with more diffuse sensory symptoms that vary over time and space and are multifaceted. Furthermore, sensitization processes involving other networks can act as additive mechanisms, associated with a combination of psychosocial stressors and a state of vulnerability that predisposes the individual to develop pain.

**Figure 1:**
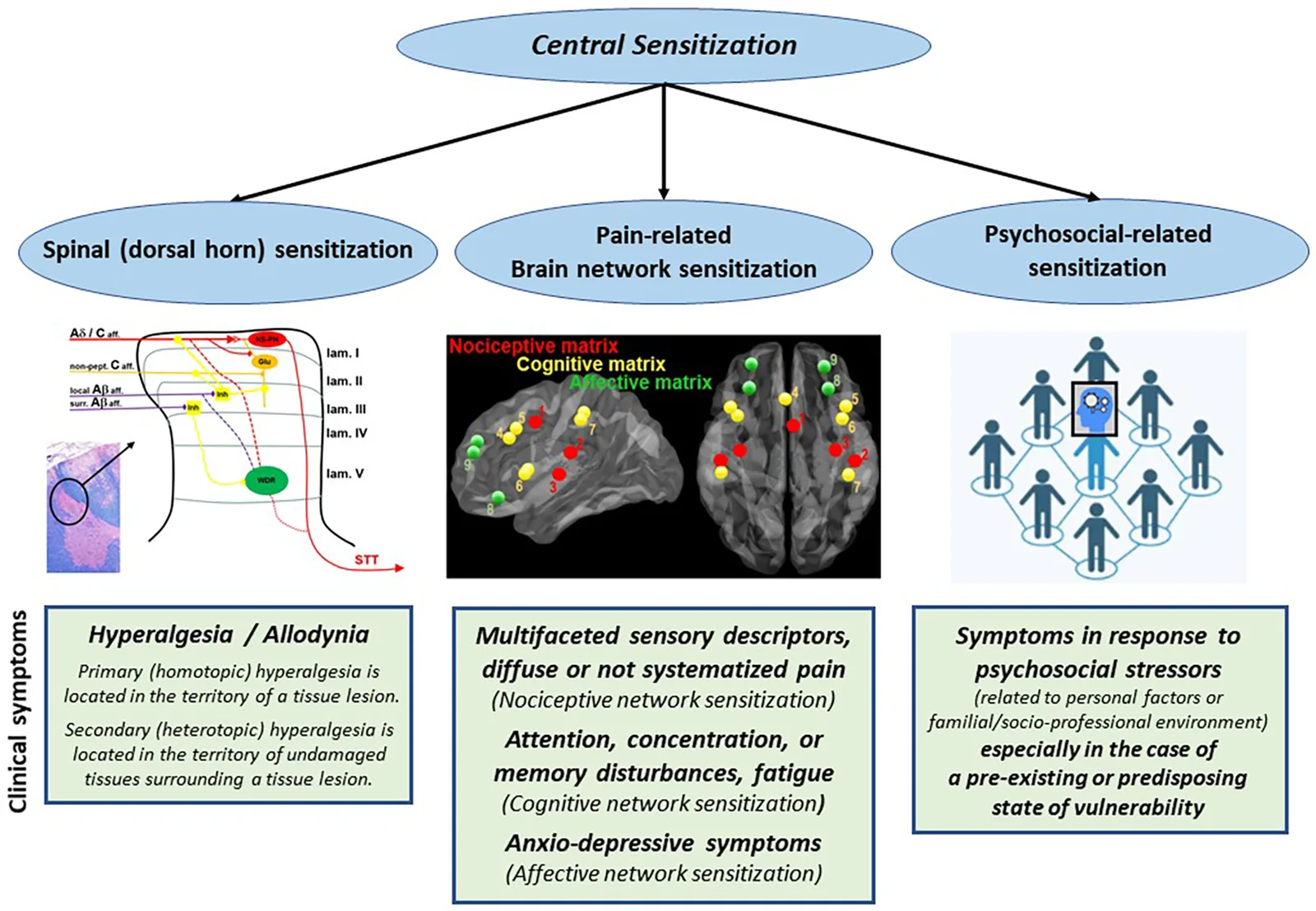
Illustration of the different types of central sensitization to pain occurring at the spinal level, involving brain networks or influenced by psychosocial factors, and associated clinical symptoms. Spinal sensitization. Small-diameter Aδ/C afferents (aff.) terminate in the most superficial laminae (lam.) of the dorsal horn, mainly in lamina I, except for non-peptidergic C fibers (non-pept.) that terminate in lamina II. These afferents connect to nociceptive-specific projection neurons (NS-PN) at the origin of the spinothalamic tract (STT) or local glutamatergic interneurons (Glu). Some Aδ afferents send collaterals to lamina V (red dashed line). Large-diameter Aβ afferents, issued from the same receptive territories as the small fibers (local) or from surrounding territories (surr.), integrate more deeply (laminae III-V), projecting to inhibitory interneurons (Inh) or wide-dynamic range neurons (WDR). Pain-related brain network sensitization. A triple-network system can be identified in pain processing, namely a nociceptive (sensory) matrix (red dots), including the mid-cingulate cortex (1), the parietal operculum (2), and the superior-posterior insula (3); a cognitive (attentional) matrix (yellow dots), including the anterior cingulate cortex (4), the dorsolateral prefrontal cortex (5), the dorsal anterior insula (6), and the posterior parietal cortex (7); an affective (emotional) matrix (green dots), including the orbitofrontal cortex (8) and the anterolateral prefrontal cortex (9). Psychosocial-related sensitization. This model suggests that pain can be sensitized by environmental psychosocial stressors, particularly given a patient’s predisposing vulnerability linked to personal history, thoughts, and beliefs, shaped by physical, emotional, cultural, and social factors. The clinical symptoms corresponding to each sensitization mechanism are indicated at the bottom of the figure.

Thus, various symptoms resulting from different pathophysiological mechanisms can be linked to a condition referred to as “central sensitization” in clinical practice: evoked pain, neuropathic-like sensory symptoms, cognitive impairment, anxiety and depressive symptoms, or psychosocial components. These different clinical elements can shed light on understanding the specific causes of a chronic pain syndrome in a given individual, and the use of a generic term like “central sensitization” only adds to the confusion. It is therefore essential to define the term “central sensitization” more precisely to better contextualize it within the clinical setting. To this end, the term “central sensitization” should be refined with more specific terms referring to a better-defined pathophysiology, such as “spinal sensitization”, “pain-related brain network sensitization”, and “psychosocial-related sensitization”, as illustrated in Figure 1. The taxonomy of chronic pain must evolve in order to characterize its mechanisms in a personalized way for each patient, a prerequisite for optimizing therapeutic strategies. Specifying the type of central sensitization mechanism is not merely a scientific or academic objective, but it also plays a critical role in clinical practice by guiding treatment choices. A patient with spinal sensitization or nociceptive brain network sensitization would not be treated in the same way as a patient with sensitization of cognitive or affective brain networks, or sensitization involving stress factors combined with psychosocial vulnerability.
